# Meshed Context-Aware Beam Search for Image Captioning

**DOI:** 10.3390/e26100866

**Published:** 2024-10-15

**Authors:** Fengzhi Zhao, Zhezhou Yu, Tao Wang, He Zhao

**Affiliations:** 1 College of Computer Science and Technology, Jilin University, Changchun 130012, China; yuzz@jlu.edu.cn (Z.Y.); taowang19@mails.jlu.edu.cn (T.W.); hezhao20@mails.jlu.edu.cn (H.Z.); 2 Key Laboratory of Symbolic Computation and Knowledge Engineering of Ministry of Education, Jilin University, Changchun 130012, China; 3 School of Data Science and Computer Science, Guangdong Peizheng College, Guangzhou 510830, China

**Keywords:** meshed context-aware, beam search, decoding strategy

## Abstract

Beam search is a commonly used algorithm in image captioning to improve the accuracy and robustness of generated captions by finding the optimal word sequence. However, it mainly focuses on the highest-scoring sequence at each step, often overlooking the broader image context, which can lead to suboptimal results. Additionally, beam search tends to select similar words across sequences, causing repetitive and less diverse output. These limitations suggest that, while effective, beam search can be further improved to better capture the richness and variety needed for high-quality captions. To address these issues, this paper presents meshed context-aware beam search (MCBS). In MCBS for image captioning, the generated caption context is dynamically used to influence the image attention mechanism at each decoding step, ensuring that the model focuses on different regions of the image to produce more coherent and contextually appropriate captions. Furthermore, a penalty coefficient is introduced to discourage the generation of repeated words. Through extensive testing and ablation studies across various models, our results show that MCBS significantly enhances overall model performance.

## 1. Introduction

In the context of image captioning [1], generating coherent, contextually appropriate captions from visual data presents unique challenges. While computers have achieved significant progress in mimicking human language generation, the underlying process fundamentally differs from how humans formulate sentences. Humans tend to conceptualize an entire sentence or phrase in their minds before selecting specific words to express it, considering the overall meaning and structure [2]. In contrast, computers generate text sequentially, producing words one by one [3], heavily relying on previously generated words to predict the next [4]. The generated caption is consequently unable to rely on global information. To address these challenges, we introduce meshed context-aware beam search (MCBS), a novel decoding strategy that dynamically integrates context during the captioning process. By adjusting attention to various image regions based on the generated context, MCBS enhances the coherence and richness of captions, improving both local and global text quality.

**Figure 1:**
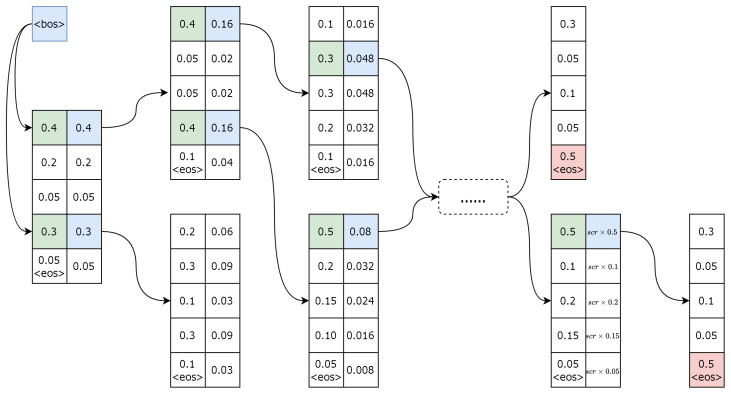
The execution process of beam search is displayed in the image.

When generating text, machines use greedy search [5], selecting the highest probability word at each step. Although simple and efficient, this often leads to local optima and lacks diversity. Top-k sampling [6] addresses this by randomly selecting a word from the top k highest probabilities, introducing more variety but sometimes reducing coherence. Nucleus sampling (top-p sampling) [3] balances diversity and quality by choosing words whose cumulative probability exceeds a threshold, though the threshold requires fine-tuning. Beam search [7], in contrast, retains the top k sequences at each step and expands them to find the highest-scoring output, offering more consistent text generation. In image captioning, where the goal is to accurately describe an image [8], decoding strategies have evolved from greedy search to more sophisticated methods like beam search [9], which explores multiple candidate sequences simultaneously. Figure 1 illustrates the fundamental process of standard beam search. Starting with the beginning-of-sequence token ‘$\langle bos\rangle$’, beam search selects the top two words at each step based on their probabilities. This selection process allows the algorithm to explore multiple candidate sequences simultaneously, enhancing the chances of generating coherent and contextually appropriate captions. The search continues until the end-of-sequence token ‘$\langle eos\rangle$’ is generated or a predefined sentence length is reached [10].

This step-by-step approach optimizes word selection based on prior words, but standard beam search has notable limitations in image captioning. While effective at local optimization, it often sacrifices global coherence for short-term gains, resulting in captions that lack the natural flow and depth of human language. In image captioning, where the goal is to describe the entire visual scene with both accuracy and richness, beam search’s tendency to prioritize local fits can result in sentences that feel disjointed or overly simplistic. Moreover, beam search heavily relies on predefined scoring functions, which tend to overemphasize high-probability word sequences, often causing the model to produce repetitive or predictable captions. This repetition not only reduces the variety of generated text but also fails to capture the full depth and nuance of an image. For example, complex visual scenes often require diverse and detailed descriptions, yet beam search may overlook alternative word sequences that better reflect the image’s richness. These limitations—repetition, narrow focus, and the lack of descriptive variation—are especially problematic in image captioning, where generating varied, coherent, and contextually rich descriptions is key to fully conveying the visual content.

To address these limitations of standard beam search in the field of image captioning, this paper introduces meshed context-aware beam search (MCBS). This approach dynamically leverages the generated textual context to influence the image attention mechanism at each decoding step. This method enhances the coherence and contextual appropriateness of generated captions by guiding the model to dynamically attend to different image regions based on contextual information as the caption is being created. It not only improves the relevance of each word to the image but also ensures that the overall caption more effectively captures the complexity and nuances of the visual content. Additionally, we introduced a penalty coefficient for sentences containing identical words, encouraging the model to express the same ideas using different words whenever possible. This approach aims to increase the diversity of generated captions. MCBS also integrates an overall landscape view of the image by dynamically updating the context vector with the attention weights from previously attended regions. This ensures that the model consistently maintains a global understanding of the image, adjusting its focus to capture not only the prominent features but also subtler aspects that contribute to a richer, more comprehensive caption. By doing so, MCBS effectively balances the local and global features, enhancing both the accuracy and fluency of the generated descriptions.

**Figure 2:**
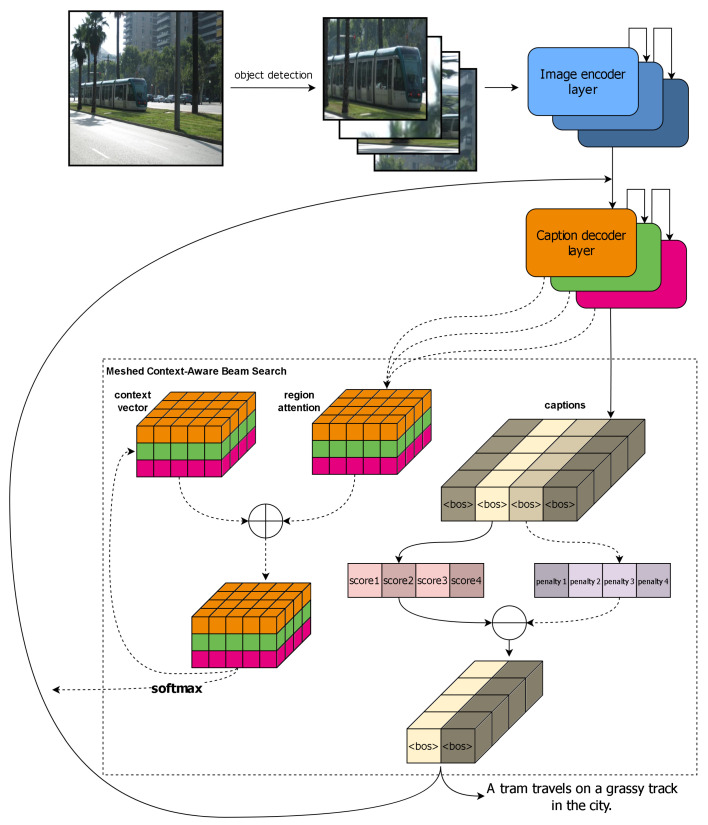
An overview of the meshed context-aware beam search.

Figure 2 illustrates the basic framework of MCBS. First, an object detection network, usually faster R-CNN, is used to detect objects in the image. Instead of outputting their specific categories, the network outputs the feature vectors of the detected regions, which are fed into the image encoding layer. These region features are encoded, and the decoder then generates the caption text based on them. Unlike standard beam search, which only considers the final output, MCBS continuously updates the attention mechanism as the caption is being generated. At each step, the decoder outputs attention weights over the image regions, which are added element-wise to a stored context vector. This produces an updated context vector that, after applying a softmax operation, is fed back into the model to guide the generation of the next word. When selecting the final caption, both the generation probability and the structure of the caption are evaluated. If repeated words are detected, a penalty factor is applied to avoid redundancy. Finally, the caption with the highest overall score is chosen, ensuring a balanced generation process that avoids repetition while maximizing fluency and relevance. In summary, the contributions of our paper are as follows:

- This paper specifically improves the standard beam search for the field of image captioning by introducing meshed context-aware beam search. This new approach is designed to address the issue where the model only focuses on a limited portion of the image, leading to more accurate and contextually relevant captions.
- This paper reviews various prior decoding strategies and beam search variants, tests their impact on the image captioning model, and analyzes the results.
- Through experimental validation, the effectiveness of the meshed context-aware beam search method is demonstrated, which not only enhances the overall performance of image captioning models, but also increases the diversity of the generated text.

In this chapter, we explain the background of the MCBS method. In Section 2, we introduce some foundational models for image captioning as well as several variants based on standard beam search. In Section 3, we provide a detailed introduction to MCBS in comparison to the standard beam search. In Section 4, we describe the dataset used, the evaluation methods applied, and other experimental details. In Section 5, we discuss the experimental results and conduct a series of ablation studies. Finally, we provide a summary of the paper in Section 6.

## 2. Related Work

In this section, we briefly reviewed the models used in image captioning and provided an introduction to some of the variants of beam search.

### 2.1. Image Captioning

Image captioning is the task of converting images into descriptive captions. Vinyals et al. [11] were among the first to propose an encoder–decoder architecture for this task, which laid the foundation for many subsequent models. The model leverages an object detection network to extract regional features from the image [12]. These regional features, along with their associated information, are encoded by the encoder into an intermediate representation [13,14], which is then decoded into a caption by the decoder. Xu et al. [15] introduced the attention mechanism into image captioning models, allowing them to dynamically focus on different parts of the image, improving the relevance and accuracy of the generated descriptions.

In 2018, Lu et al. [16] proposed the neural baby talk model, which integrates a module to produce object-level descriptions before generating natural language sentences. This approach enables the model to first understand and identify objects within an image, resulting in more accurate and contextually relevant captions. The same year, the introduction of the Transformer architecture [17] marked a significant advancement in the field of image captioning. Parmar et al. [18] were the first to experiment with the attention mechanism in this context, and their results demonstrated that it significantly outperformed traditional LSTM-based models. Building on this, Cornia et al. [19] utilized the Transformer and introduced a meshed connectivity pattern combined with memory-augmented attention mechanisms. This design greatly enhanced the model’s ability to generate detailed and contextually rich captions by efficiently integrating and referencing both visual and semantic information. Later, Luo et al. [20] proposed the dual-level collaborative transformer for image captioning, which utilizes a collaborative attention mechanism to fuse global and local features, leading to significant improvements in the accuracy and richness of the generated captions. Recently, attention mechanisms have been applied in various image processing tasks. For example, adaptive graph attention has been used in blind image quality assessment to enhance local and contextual information, improving accuracy [21]. A multi-scale attention network with large kernel attention and gating mechanisms has also been applied to single-image super-resolution for better restoration [22]. Additionally, transformers with optical flow prediction have been used for video frame interpolation to generate intermediate frames more efficiently [23], while another approach eliminates optical flow entirely, relying on deep learning for direct frame prediction [24]. Additionally, many other innovative methods [25,26,27] have been proposed in recent years, collectively advancing the field of image captioning.

### 2.2. Decoding Strategy

Beam search is a widely used decoding strategy in sequence generation tasks [28], but it has several limitations such as a greedy strategy, high computational complexity, and a lack of diversity. Various methods [29] have been proposed to address these issues. Diverse beam search [7] introduces a diversity penalty to ensure that the generated sequences are more distinct from each other, thereby increasing text diversity. Length penalty beam search balances the scores of longer and shorter sequences by penalizing sequences based on their length, addressing the bias towards shorter sequences in standard beam search. Soft alignment beam search [30] incorporates soft attention mechanisms into the beam search process, ensuring more effective alignment between the generated sequence and the input, which improves the utilization of relevant context and enhances sequence quality. Lookahead beam search [9] considers future steps’ scores in each step, enhancing the quality of current decisions by estimating the potential of future steps.

To address the limitations of standard beam search, such as its tendency to favor shorter sequences, lack of diversity, and computational inefficiency, various improvements have been proposed. Bayesian beam search [31] introduces uncertainty estimates into the search process, allowing the model to guide the exploration based on the probability distribution’s uncertainty, thereby improving the search’s robustness. Hybrid beam search combines multiple search strategies to take advantage of their respective strengths, offering a more flexible and effective decoding approach. Similarly, adaptive beam search [32] dynamically adjusts the beam width during decoding based on intermediate results and scores, optimizing both computational efficiency and output quality. To address the risk of local optima, Monte Carlo beam search [33] introduces randomness by sampling multiple candidate words at each step, promoting exploration in the search space. Global normalization beam search [34] addresses the bias towards shorter sequences by considering the overall sequence score rather than focusing on local decisions at each step. Related to this, minimum Bayes risk beam search [35] minimizes the expected risk rather than simply maximizing probability, generating sequences that are more aligned with practical application needs. Softmax temperature scaling beam search [17] controls sequence diversity by adjusting the temperature parameter of the softmax function, allowing more balanced probability distributions depending on the task’s needs. Dynamic beam allocation further enhances resource efficiency by dynamically adjusting the beam width based on the sequence scores and diversity considerations during the decoding process. Additionally, length normalization beam search [36] compensates for the bias towards shorter sequences by normalizing scores based on sequence length, ensuring fairer comparisons among outputs. Lastly, minimum risk beam search [37] optimizes the generated sequences by directly minimizing expected risk, leading to outputs that better align with specific evaluation metrics.

These methods collectively enhance beam search by improving diversity, efficiency, and sequence quality, making them more suitable for a variety of sequence generation tasks.

## 3. Method

Previous improvements have primarily focused on text generation tasks and have not been specifically designed for image captioning. In this section, we introduce an MCBS method that is optimized for the task of generating image captions.

### 3.1. Standard Beam Search

The standard beam search not only considers the probability of each word at every generation step but also takes into account the probability of the entire sentence.

Algorithm 1 initializes the beam with the token ‘$\langle bos\rangle$’ and a score of 0. The beam serves as a pool of candidate sequences. The loop runs until all sequences end with ‘$\langle eos\rangle$’ or reach the maximum length. For each sequence, if it does not end with ‘$\langle eos\rangle$’, the model predicts the next word probabilities using the current sequence and image features. New sequences are generated by appending words from the vocabulary, with scores updated using the log-probability of the word. After expanding all sequences, the top ‘beam_width‘ sequences are selected for the next iteration.
**Algorithm 1** Standard Beam Search1:**function** standardbeamsearch(model, image, beam_width)2:    beams ← [([$\langle bos\rangle$], 0)]3:    **while** not all beams end with $\langle eos\rangle$ and beams_len < max_len **do**4:        new_beams ← [ ]5:        **for** each (seq, score, context) in beams **do**6:           **if** seq ends with $\langle eos\rangle$ **then**7:               new_beams.append((seq, score))8:           **else**9:               vocab ←$\left\{\left(w,p_{w}\right)|p_{w}=model\left(image,seq\right)\right\}$10:               **for** each $\left(w,p_{w}\right)$ in vocab **do**11:                   new_seq ← seq + *w*12:                   new_score ← score + log($p_{w}$)13:                   new_beams.append((new_seq, new_score))14:               **end for**15:           **end if**16:        **end for**17:        beams ← top beam_width from new_beams based on score18:    **end while**19:    **return** the seq with the highest score from beams20:**end function**

The process continues until all sequences end with $\langle eos\rangle$ or reach the maximum length limit. The highest-scoring sequence is returned. While beam search boosts performance, it has limitations, such as focusing too narrowly on high-scoring sequences without considering broader context, often leading to repetitive outputs that reduce diversity and creativity in the generated text.

### 3.2. Meshed Context-Aware Beam Search

In MCBS, the most crucial component is the context memory, which stores the attention given to different regions of the image based on the generated caption so far. Initially, it is set as a vector of zeros and stored in the beam along with the ‘$\langle bos\rangle$’ token and an initial caption generation probability of 0. The model’s output at each step relies not only on the image features and the already generated sequence, but also generates the current attention for the context.

#### 3.2.1. Context Vector

The context vector is a dynamic representation that encapsulates the accumulated attention or focus on the different regions of an image as a text sequence is being generated. It serves as a memory that evolves with each word generated, integrating both the visual features of the image and the partial text sequence produced so far. Initially, the context vector is typically initialized as a zero vector, indicating no prior focus. It is stored in the beam along with the word sequence and the probability of the generated annotation.

(1) $$beams\leftarrow [\left(\left[\langle bos\rangle \right],0,context\right)].$$

When generating a caption, the context vector is first normalized by applying a softmax function, as shown in Equation (2). The normalized context vector (attn) is then combined with the image feature vectors and the partially generated caption sequence, which are all input into the model together, as described in Equation (3). This approach ensures that the context is consistently accounted for during the generation process, allowing the model to produce more coherent and contextually relevant outputs. The normalization of the context vector helps maintain a balanced influence of different contextual elements, leading to more accurate predictions and improved caption generation quality:

(2) $$\begin{array}{cc} attn & \leftarrow \mathrm{SOFTMAX}(context), \end{array}$$

(3) $$\begin{array}{cc} vocab & \leftarrow \{\left(w,p_{w},\mathrm{new\_attn}\right)\leftarrow \mathrm{MODEL}\left(image,seq,attn\right)\}. \end{array}$$

After a word is generated, the context vector is updated at each step to reflect the model’s shifting attention based on the evolving textual context. As shown in Equation (3), the new context is obtained by adding the newly calculated attention (‘new_attn’) to the existing context. This continuous update allows the model to adapt its focus dynamically, ensuring that the generated text remains contextually relevant and coherent with respect to the image:

(4) new_context←context+new_attn.

#### 3.2.2. Attention in the Model

The attention vector is adjusted so that its components sum to one, typically achieved using a softmax function. This normalization process transforms raw context values into a probability distribution, enabling the model to appropriately weigh each contextual element. By normalizing the context features, the model can better balance the influence of different parts of the context, preventing any single element from dominating the decision-making process during text generation. This leads to more accurate and contextually aligned outputs. The context vector is related to the image features, with dimensions corresponding to the number of regional features in the image. We use a fully connected layer model to adaptively adjust the attention weights:

(5) middle_attn=fc(1−attn),

where fc represents the fully connected layer. In the standard beam search model, caption generation relies on the attention mechanism, where *Q* represents the previously generated sequence, and *K* and *V* represent the image features. The final attention output is converted into a probability distribution over the words, as shown below:

(6) $$\begin{array}{cc} Q=X_{q}W_{q},K & =X_{k}W_{k},V=X_{v}W_{v},\\ \Omega_{A} & =\mathrm{Softmax}\left(\frac{QK^{⊤}}{\sqrt{D}}\right),\\ Att & =\Omega_{A}V. \end{array}$$

In the MCBS model, the calculation method of $\Omega_{A}$ has been modified. When computing the attention, it is element-wise multiplied with the previously obtained middle_attn:

(7) $$\Omega_{A}=\mathrm{Softmax}\left(\frac{QK^{⊤}}{\sqrt{D}}\times \mathrm{middle\_attn}\right).$$

The newly generated $\Omega_{A}$ further adjusts the current attention weights, ensuring that it not only considers the current image features but also incorporates the previous attention distribution. This adjustment allows the model to maintain a more consistent focus on relevant image regions, preventing unnecessary shifts in attention during the decoding process. By returning the newly generated $\Omega_{A}$ as new_attn, the model uses this updated attention matrix to guide the generation of the next word in subsequent steps. This process helps produce more coherent and contextually consistent text descriptions.

#### 3.2.3. Repetition Penalty

In many sequence generation tasks, particularly in image captioning, one of the common challenges is the occurrence of repetitive phrases or words, which can reduce the quality and expressiveness of the generated output. To address this issue, we introduce a repetition penalty mechanism that specifically targets the reduction in redundant words within the generated sequence.

The repetition penalty is computed dynamically as the sequence is generated. At each step, the penalty is applied to the score of any word that has already appeared in the sequence. The overall penalty for a given sequence is the sum of penalties applied to all repeated words. This is formalized by the following equation:

(8) $$\mathrm{RepPen}\left(\mathrm{new\_seq}\right)=\sum_{w\;\in \;\mathrm{new\_seq}}\max\left(0,\left(n_{w}-1\right)\times \mathrm{pen\_coef}\right),$$

where $n_{w}$ represents the number of occurrences of the word *w* in the sequence new_sequence, and penalty_coef is a predefined penalty coefficient. For each word *w* that appears more than once in the sequence, the penalty is calculated as $(n_{w}-1)\times \mathrm{penalty\_coef}$. This means that, for a word appearing twice in the sequence, the penalty would be equal to penalty_coef, and for a word appearing three times, the penalty would be twice penalty_coef, and so on. If a word appears only once, the penalty is zero.

During the decoding process, the penalty is subtracted from the log-probability score of the sequence. This adjustment reduces the overall score of sequences with repeated words, making them less likely to be selected as top candidates in the subsequent steps of beam search or other decoding strategies. As a result, the model is encouraged to explore alternative word choices that lead to more varied and semantically rich captions. In summary, the repetition penalty is a crucial component of our decoding strategy, addressing the challenge of redundancy in sequence generation. By incorporating this mechanism, we significantly enhance the quality of the generated text, making it more diverse, coherent, and expressive.

#### 3.2.4. Overall Architecture

This section introduces the meshed context-aware beam search algorithm, designed to enhance image captioning by dynamically adjusting attention. Algorithm 2 presents the overall operational logic of MCBS. Unlike standard beam search, it uses both image features and generated captions to guide the prediction of the next word. By updating the attention vector and applying a repetition penalty, the algorithm ensures more coherent and diverse captions.

The code provided outlines the implementation of the meshed context-aware beam search algorithm specifically designed for image captioning. This method begins by initializing with a single beam containing the start-of-sequence token, where both the initial generation probability and the attention vector are set to zero. The main loop of the algorithm continues until all candidate sequences end with the end-of-sequence token or the maximum caption length is reached. During each iteration, the algorithm expands the current sequences by predicting the next word in the caption.

For sequences that have not yet reached the end-of-sequence token, the context feature vector is first normalized to obtain the attention vector. Unlike standard beam search, MCBS takes not only the image features and the previously generated text as inputs but also includes the attention vector. This attention vector guides the model’s focus to different regions of the image, thereby influencing the prediction of the next word. After generating each word, it is appended to the existing sequence of captions. The sequence’s generation probability is then adjusted, where MCBS applies a repetition penalty to reduce the likelihood of generating captions with repeated words, thus promoting diversity in word choice. Finally, the new attention vector is integrated into the context vector. The updated sequence, probability, and context vector are then added to the new set of beams.
**Algorithm 2** Meshed Context-Aware Beam Search1:**function** meshedcontextaware(model, image, beam_width, penalty)2:    beams ← [([$\langle bos\rangle$], 0, context)]3:    **while** not all beams end with $\langle eos\rangle$ and beams_len < max_len **do**4:        new_beams ← [ ]5:        **for** each (seq, score, context) in beams **do**6:           **if** seq ends with $\langle eos\rangle$ **then**7:               new_beams.append((seq, score, context))8:           **else**9:               attn ← softmax(context)10:               vocab $\leftarrow \{\left(w,p_{w},\mathrm{new\_attn}\right)\leftarrow$ model(image, seq, attn)}11:               **for** each $\left(w,p_{w},\mathrm{new\_attn}\right)$ in vocab **do**12:                   new_seq ← seq + *w*13:                   rep_penalty ← penalty × reppenalty(new_seq)14:                   new_score ← score + log($p_{w}$) - repetition_penalty15:                   new_context ← context + new_attn16:                   new_beams.append((new_seq, new_score, new_context))17:               **end for**18:           **end if**19:        **end for**20:        beams ← top beam_width from new_beams based on score21:    **end while**22:    **return** the sequence with the highest score from beams23:**end function**

After calculating the new sequences for all possible extensions, the algorithm only retains the top candidate sequences based on their cumulative scores, as determined by the beam width parameter. This process continues iteratively, with each step focusing on refining the captions by dynamically adjusting the attention based on the generated context and penalizing repetition. Once the process is complete, the sequence with the highest cumulative score is returned as the final caption for the image. This approach ensures that the generated captions are not only relevant to the image but also coherent and diverse, effectively addressing the limitations of traditional beam search in image captioning tasks.

## 4. Experiment

In this section, we introduce the experimental details of image captioning in this paper. In Section 4.1, we describe the datasets and evaluation methods used in our experiments. Section 4.2 covers the training steps. In Section 4.3 and Section 4.4, we introduce several decoding strategies and standard beam search variants as comparison methods. In Section 4.5, we briefly introduce several models used for testing decoding strategies.

### 4.1. Dataset and Evaluation Methods

**Dataset:** The MSCOCO dataset [38] is a richly annotated dataset featuring over 330,000 images and 1.5 million object instances, designed for tasks such as object detection and image captioning. Unlike earlier datasets that focus on isolated objects, MSCOCO emphasizes the context in which objects appear, making it especially useful for tasks that require understanding complex scenes. Each image includes multiple descriptive captions, providing a valuable resource for generating human-like descriptions. Its diversity and detailed annotations present a significant challenge for models, requiring them to grasp intricate relationships within scenes to produce contextually appropriate captions.

We selected the MSCOCO dataset for our image-captioning experiments because of its diverse images and high-quality human-annotated captions, offering a rich source of visual and textual data. Each image is paired with five captions, making the dataset ideal for evaluating the generalization and robustness of our proposed method, meshed context-aware beam search (MCBS). Additionally, MSCOCO’s detailed annotations facilitate learning rich contextual relationships, improving both the accuracy and quality of generated captions. This makes it an excellent benchmark for testing our method’s ability to generate coherent, contextually rich captions across various types of image content.

**Evaluation methods:** Evaluating image captioning models involves comparing generated captions against reference captions using various metrics that assess the different aspects of the text’s quality. The most commonly used metrics in this field are BLEU, METEOR, ROUGE, and CIDEr [39,40,41,42], each of which offers unique insights into the generated captions’ performance. BLEU measures n-gram precision, focusing on how closely the generated text aligns with reference captions in terms of word choices and phrasing, though it sometimes overlooks semantic meaning. METEOR complements BLEU by accounting for synonyms and stemming, providing a more flexible evaluation of content overlap and meaning. ROUGE, primarily a recall-based metric, evaluates how much of the reference content is captured in the generated caption. Lastly, CIDEr gives higher weight to words and phrases that appear across multiple reference captions, making it particularly suitable for image captioning, where accurately describing visual content is critical. Using these complementary metrics ensures a more comprehensive evaluation of the generated captions, covering both accuracy and fluency.

- BLEU (Bilingual Evaluation Understudy) is a precision-based metric that compares n-grams in the generated caption to those in the reference captions. It is defined as:
  (9) $$\mathrm{BLEU}=\exp\left(\sum_{n=1}^{N}w_{n}\log p_{n}\right)\cdot \min\left(1,\exp\left(1-\frac{{\mathrm{reference}}_{\mathrm{length}}}{{\mathrm{generated}}_{\mathrm{length}}}\right)\right),$$
  where $p_{n}$ is the precision of n-grams, $w_{n}$ is the weight for n-gram precision, typically $\frac{1}{N}$, ${\mathrm{generated}}_{\mathrm{length}}$ is the length of the generated caption, and ${\mathrm{reference}}_{\mathrm{length}}$ is the length of the reference caption. BLEU is a metric where higher values indicate better performance.
- METEOR (Metric for Evaluation of Translation with Explicit Ordering) evaluates image captions based on precision, recall, and a harmonic mean (F-score) that accounts for synonyms, stemming, and paraphrasing. In image captioning, precision measures the overlap of unigrams in the generated caption with the reference caption, while recall measures how many of the reference unigrams appear in the generated caption. It is defined as:
  (10) $$\;\mathrm{METEOR}\;=\frac{10\cdot P\cdot R}{R+9\cdot P}\cdot \left(1-\;\mathrm{penalty}\;\right),$$
  where *P* is the precision of unigram matches, and *R* is the recall of unigram matches. The penalty term accounts for the degree of fragmentation in the matched unigrams, which can penalize captions that are too disjointed or lack fluency.
- ROUGE (Recall-Oriented Understudy for Gisting Evaluation) measures the longest common subsequence (LCS) between the generated and reference captions, focusing on both the recall and precision of the LCS. The ROUGE score is computed as the F-measure between LCS precision and LCS recall, and is defined as:
  (11) $$\mathrm{ROUGE}=\frac{\left(1+\beta^{2}\right)\cdot \mathrm{LCS}-\mathrm{Precision}\cdot \mathrm{LCS}-\mathrm{Recall}}{\beta^{2}\cdot \mathrm{LCS}-\mathrm{Precision}+\mathrm{LCS}-\mathrm{Recall}},$$
  where β is typically set to 1, LCS−Precision is the precision of the longest common subsequence, and LCS−Recall is the recall of the longest common subsequence. Specifically, for image captioning: LCS−Precision is the ratio of the length of the longest common subsequence to the length of the generated caption. LCS−Recall is the ratio of the length of the longest common subsequence to the length of the reference caption.
- CIDEr (Consensus-Based Image Description Evaluation) is specifically designed for image captioning. It measures the similarity between generated captions and reference captions using term frequency-inverse document frequency (TF-IDF) weighting for n-grams. The CIDEr score is calculated as:
  (12) $$\;\mathrm{CIDEr}\;=\frac{1}{m}\sum_{i=1}^{m}\sum_{n=1}^{N}w_{n}\cdot \;{\mathrm{CIDEr}}_{n},$$
  where *m* is the number of reference captions, *N* is the maximum n-gram length (usually up to 4), $w_{n}$ is the TF-IDF weight for the n-gram, and ${\mathrm{CIDEr}}_{n}$ measures the similarity of n-grams of length *n* between the generated and reference captions.

Each metric provides a different perspective on the quality of the generated captions. BLEU focuses on n-gram precision, METEOR incorporates semantic meaning, ROUGE emphasizes the longest common subsequence, and CIDEr uses TF-IDF weighting for a more context-aware comparison. Using these metrics together helps ensure a comprehensive evaluation of image captioning models, capturing both the syntactic and semantic aspects of the generated text.

### 4.2. Training Steps

Image captioning models are typically trained in two steps: cross-entropy (CE) training and reinforcement learning (RL) with self-critical sequence training (SCST). In the first step, the model is trained using the cross-entropy (CE) loss. This approach treats image captioning as a sequence prediction problem, where the model generates a caption word by word. The CE loss for a given target sequence $\left(y_{1},y_{2},\ldots ,y_{T}\right)$ is defined as:

(13) $$L_{\mathrm{CE}}=-\sum_{t=1}^{T}\log P\left(y_{t}|y_{1:t-1},I\right),$$

where *T* is the length of the target sequence, $y_{t}$ is the ground truth word at position *t*, and $\log P\left(y_{t}|y_{1:t-1},I\right)$ is the probability of the word $y_{t}$ given the previous words $y_{t-1}$ and the image *I*. This loss function encourages the model to generate captions that closely match the ground truth by minimizing the negative log-likelihood of the correct words at each time step. After initial training with CE loss, the model is fine-tuned using reinforcement learning (RL) with self-critical sequence training (SCST) and the sentences are generated by a pre-trained model without using real annotations. This method optimizes a sequence-level metric directly by treating the problem as a reinforcement learning task. The reward $r(\hat{y})$ is calculated based on the quality of the generated sequence $\hat{y}$ and the policy gradient loss is defined as:

(14) $$L_{\mathrm{RL}}=-\left(r\left(\hat{y}\right)-b\right)\sum_{t=1}^{T}\log P\left({\hat{y}}_{t}|{\hat{y}}_{1:t-1},I\right),$$

where $\hat{y}$ is the sequence generated by the model, $r(\hat{y})$ is the reward for the generated sequence, typically a metric like CIDEr. *b* is a baseline reward, usually the reward obtained by greedy decoding to reduce variance in the gradient estimation. The SCST approach uses the difference between the reward of the sampled sequence and the baseline reward to update the model, encouraging it to generate sequences with higher rewards.

By first training the model with cross-entropy loss to learn the basic captioning structure and then fine-tuning with reinforcement learning to directly optimize for sequence-level metrics, we can achieve more accurate and human-like image captions. The combination of these two steps leverages the strengths of both supervised learning and reinforcement learning to improve the overall performance of image captioning models.

### 4.3. Different Decoding Strategy

In this section, we introduced different decoding strategies in Table 1, including Greedy Search, Top-k, and nucleus sampling (Top-p). In the subsequent ablation experiments, we compared these strategies with the standard beam search.

To conclude this section, our exploration of different decoding strategies highlights the unique strengths and weaknesses of each approach. While greedy search provides a straightforward and efficient method, it often sacrifices diversity for simplicity. Top-k and nucleus sampling offer more flexibility, allowing for a broader range of outputs, but they can introduce variability that may not always lead to the most coherent results. In the subsequent ablation experiments, we will demonstrate how these methods compare to the standard beam search.

### 4.4. Beam Search Variants

In this section, we introduce several classic beam search variants. These variants have been developed to address specific limitations of the standard beam Search algorithm, offering improvements in various aspects such as diversity, efficiency, and overall performance in text generation tasks.

- Diverse beam search (DBS) addresses standard beam search’s tendency to generate similar sequences by introducing a diversity penalty. This encourages more distinct outputs, enhancing the variety and richness of the generated sequences.
- Length penalty was introduced to counter beam search’s bias towards shorter sequences. By normalizing scores based on sequence length, this method encourages longer, more complete outputs, ensuring a fairer comparison between sequences of different lengths.
- Soft alignment penalty tackles uneven attention distribution in sequence generation. By penalizing overly concentrated attention on specific input parts, it promotes more balanced focus across the entire input. This leads to more coherent and representative outputs, with the penalty strength fine-tuned for optimal alignment.
- Lookahead addresses the short-sightedness of standard beam search by evaluating potential future steps. This helps the model make more informed decisions, improving the overall quality of generated sequences and avoiding poor early-stage choices.

In summary, these beam search variants each address specific limitations of the standard algorithm, providing enhancements in terms of diversity, length balance, attention distribution, and forward-looking decision making. By incorporating these improvements, text generation tasks can achieve higher-quality outputs that are not only more varied but also more efficient and aligned with the intended goals of the task. These advancements collectively push the boundaries of what can be achieved in sequence generation, making these variants valuable tools for a wide range of natural language processing applications. However, these methods are designed for different fields and have not been specifically tailored for image captioning. Subsequently, we tested their performance in the field of image captioning.

### 4.5. Evaluation Models

To validate the performance of various decoding strategies, including different variants of beam search and newly proposed beam search methods, we conducted experiments using multiple image captioning models. These experiments were designed to thoroughly evaluate the effectiveness and limitations of each decoding approach across different model architectures. By comparing the outputs generated by each method, we aimed to gain insights into how these strategies influence the quality, diversity, and accuracy of the captions produced, ultimately identifying the most effective decoding strategy for image captioning tasks.

The models used include the image transformer (Trans) [18], which utilizes a transformer architecture to convert images into descriptive text, and the meshed-memory transformer ($M^{2}$) [19], which enhances the former by incorporating memory mechanisms for better context understanding. The attention on attention (AoA) [43] model improves the focus on important image areas by refining the attention mechanism, while the dual-level collaborative transformer (DLCT) [20] employs two levels of attention to balance global and local details in captions. Lastly, the beyond a pre-trained object detector (BPOD) model [44] leverages a cross-modal context to generate captions that consider both visual and textual information, not just detected objects. By applying these models, we assessed how different decoding strategies impact the quality of the generated captions.

## 5. Results and Ablation Study

This section tests the performance of the MCBS to validate its effectiveness. We also conducted experiments on existing decoding strategies and beam search-based variants. Subsequently, we separately evaluated the two components of MCBS, and then assessed the performance of MCBS with different beam sizes, comparing it with standard beam search. Finally, we examined the performance of MCBS across different datasets.

### 5.1. Results

**Figure 3:**
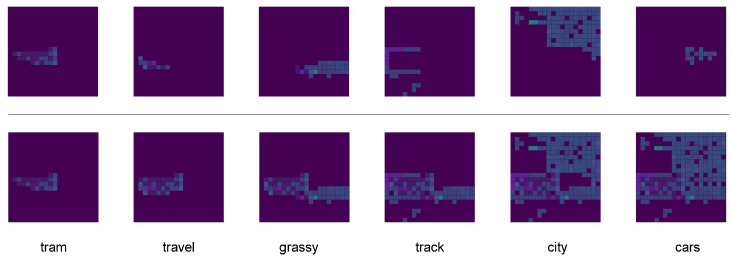
The examples of the new attention (new_att) and context vector focus in images.

In Figure 3, we visualize the new attention (new_att) and context vector. The figure shows the level of attention that the model pays to the specific areas of the image while generating the corresponding words. As the caption is progressively generated, the context vector gradually incorporates the overall content of the image, enabling a more holistic understanding of the scene. This visualization highlights how attention shifts and expands its focus throughout the caption generation process.

The experimental results presented in Table 2 highlight the effectiveness of the proposed MCBS compared to the standard beam search across five different image captioning models: Trans, M², AoA, DLCT, and BPOD. Each model was evaluated using a variety of metrics, including BLEU-1 (B1), BLEU-4 (B4), METEOR (M), ROUGE (R), and CIDEr (C), which collectively assess the quality, fluency, and relevance of the generated captions.

Each evaluation metric in image captioning serves a distinct purpose. BLEU measures how closely the generated captions align with reference captions by comparing n-grams, with higher scores indicating better word and phrase accuracy. METEOR goes beyond exact matches by accounting for synonyms and stems, evaluating semantic similarity. ROUGE focuses on recall, assessing how much of the reference caption’s content is captured in the generated text. CIDEr, designed specifically for image captioning, measures consensus with human descriptions by giving more weight to commonly used phrases, ensuring that the captions are both relevant and natural. Together, these metrics offer a comprehensive view of caption quality, covering accuracy, fluency, and content diversity.

Across all models, the MCBS consistently yields better results on all metrics. For instance, in the image transformer model, there is a notable improvement in the CIDEr score from 126.4 to 129.2, reflecting a significant enhancement in how well the generated captions align with human descriptions. The BLEU-1 score also increased from 80.6 to 81.9, indicating a better match of the generated n-grams with the reference captions. Similarly, in the $M^{2}$, the CIDEr score increased from 128.7 to 130.4, and BLEU-4 saw an increase from 39.1 to 40.4. These improvements suggest that the MCBS enables the model to generate captions that are not only more accurate but also more contextually relevant, as evidenced by the higher METEOR and ROUGE scores. The AoA model, which focuses on refining attention mechanisms, also benefits from the MCBS, with its CIDEr score improving from 129.8 to 131.4. This indicates that the model is able to generate captions that are more closely aligned with human descriptions, capturing both the global and local aspects of the image more effectively.

Notably, the DLCT shows only a modest improvement, with its CIDEr score increasing from 133.8 to 134.7. This is likely because DLCT effectively integrates grid features from the image during encoding, enabling the model to already capture a comprehensive representation of the image. Similarly, the BPOD model, which leverages cross-modal context, shows consistent improvement across all metrics when applying MCBS. The CIDEr score increased from 135.9 to 136.4, and the BLEU-1 score improved from 81.5 to 82.9. However, since BPOD also partitions the image into regions while maintaining a global focus, the improvement is not as substantial as with other methods. These results demonstrate the effectiveness of MCBS in enhancing the overall quality and diversity of generated captions across different models.

In summary, the experimental results clearly demonstrate that the MCBS outperforms the standard beam search across various models and metrics. This method not only improves the relevance and diversity of the generated captions but also ensures that they are more contextually aligned with the visual content, effectively addressing the limitations of traditional beam search in image captioning tasks.

### 5.2. Different Decoding Strategies

Decoding strategies influence image captioning by balancing fluency and diversity. Greedy search generates fluent but often generic captions by picking the highest probability words, while beam search explores multiple sequences for better coherence but may lack diversity. Top-k sampling (k=5) increases variety by considering the top probable words, and nucleus sampling (p=0.5) further balances fluency and diversity by sampling from a dynamic word subset. In short, greedy and beam search focus on fluency, while Top-k and nucleus sampling enhance diversity, sometimes at the cost of coherence.

The performance of various models under different decoding strategies is presented in Table 3. As shown in the table, under the cross-entropy training method, the performance of greedy search and beam search is nearly identical. This is due to the nature of cross-entropy training, where both strategies generate the next word based on the previous true word. However, there is a significant difference between the two decoding strategies when trained with reinforcement learning. Greedy search consistently selects the most optimal word, while beam search evaluates the overall performance of the sentence. The table also reveals a substantial performance drop for Top-k sampling and nucleus sampling, especially in terms of CIDEr scores, under both training methods. These two strategies introduce randomness into the decoding process by selecting the next word based on a probability distribution rather than strictly following the highest probability. While this can produce more diverse and creative outputs, it also increases the risk of generating less coherent or contextually relevant captions, leading to lower CIDEr scores. These methods may produce captions that are plausible but not necessarily the most accurate or contextually appropriate for the given image, resulting in lower alignment with the ground truth captions.

### 5.3. Results of Beam Search Variations

In the field of image captioning, we evaluated different beam search variants to address common challenges like repetition, lack of diversity, and ensuring coherence in generated captions. As shown in Table 4, we tested diverse beam search, length penalty, soft alignment penalty, and lookahead strategies, each designed to tackle the specific limitations of standard beam search. These variants aim to improve image captioning by introducing mechanisms that enhance the quality and diversity of the captions, such as discouraging repetition or accounting for future steps in sequence generation. However, the experimental results show minimal improvement over standard beam search, likely due to the high precision required in image captioning tasks where capturing the exact context of the image is critical. Thus, while these methods offer potential benefits in other domains, they do not significantly enhance the image captioning performance in our tests.

One reason for the lack of improvement could be that these alternative methods, while addressing specific issues like diversity or fluency, may introduce trade-offs that dilute the overall effectiveness in capturing the most contextually relevant and accurate captions. The additional modifications introduced by these other techniques, such as promoting diversity or adjusting for length, may introduce complexity that does not necessarily translate into better alignment with the ground truth captions, especially in a domain like image captioning where the precision of word choice is critical. Secondly, while these methods aim to address specific issues—like avoiding repetitive outputs or improving sentence fluency—they may inadvertently disrupt the balance that standard beam search maintains between relevance and coherence. For example, diverse beam search might generate more varied captions, but this diversity can sometimes lead to less accurate descriptions of the image. Similarly, penalties and lookahead strategies, while designed to improve the specific aspects of the generation process, might not align well with the specific demands of image captioning, where a delicate balance between accuracy, relevance, and fluency is essential. In summary, the additional complexity introduced by these methods does not necessarily translate into a better performance in image captioning, where standard beam search already performs well by focusing on the most likely and contextually appropriate sequences.

### 5.4. Different Components

We evaluated the performance of the model with different components. Table 5 summarizes the results across several evaluation metrics: B1, B4, METEOR, ROUGE, and CIDEr. The table compares the effects of incorporating context information and a repetition penalty on the model’s outputs.

Based on the results presented in Table 5, we can observe the impact of the ‘context‘ and ‘penalty‘ components on the model’s performance. BLEU-1 (B1) measures how well individual words in the generated captions align with reference captions, capturing word-level precision. The baseline model (Trans) achieves a solid B1 score of 80.6, indicating a good alignment of individual words. BLEU-4 (B4), which looks at 4-word sequences (n-grams), provides insight into the fluency and coherence of the generated sentences. The addition of context improves B4 from 38.8 to 39.9, suggesting that incorporating contextual information allows the model to generate more fluent and coherent sentences. METEOR goes beyond exact word matches by accounting for synonyms and stemming, offering a broader evaluation of semantic accuracy. A higher METEOR score means that the generated captions are semantically closer to the reference captions. The baseline model starts at 28.6, and this score increases to 29.1 with the full model (context + penalty), showing that both components enhance the semantic quality of the generated captions. ROUGE primarily measures recall, evaluating how much of the reference caption’s content is captured in the generated text. A higher ROUGE score reflects better coverage of the important aspects of the reference text. The model’s ROUGE score improves from 58.3 to 59.1, suggesting that the full model captures a broader range of relevant content from the image. Finally, CIDEr, specifically designed for image captioning tasks, measures the consensus between generated and reference captions by giving more weight to phrases that occur frequently across multiple references. The CIDEr score is particularly important for evaluating how well the captions reflect the human consensus. The score improves from 126.4 in the baseline model to 129.2 with both context and penalty, indicating that these components help the model generate more human-like and descriptive captions. These results demonstrate that context significantly improves caption accuracy and relevance, while penalty enhances fluency and diversity. Together, they lead to the best overall performance across all metrics.

### 5.5. Different Beam Size

In this ablation study, we examine the impact of different beam sizes [45] on the performance of image captioning models. Beam size is an important factor in balancing computational efficiency and caption quality. We test a range of beam sizes, evaluating the captions using metrics to assess fluency, relevance, and diversity. This analysis provides insights into optimizing beam size for improved image captioning performance.

**Figure 4:**
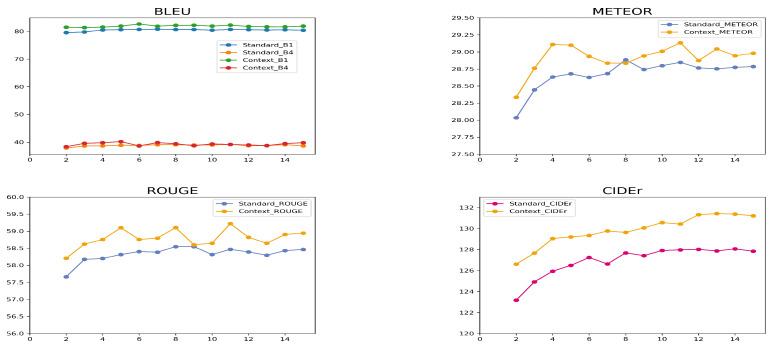
The performance of the model under different beam sizes. During the reinforcement learning training, the *b* in Equation (14) is a baseline reward, which is the average score of the output sentences. When the beam size is 1, $r(\hat{y})-b=0$ cannot be used to calculate the loss, so the beam size starts from 2.

Figure 4 shows us the experimental results of BLEU, METEOR, ROUGE, and CIDEr under different beam sizes. The experimental results demonstrate that MCBS consistently outperforms standard beam search across all metrics. While the BLEU scores remain relatively stable for both methods, MCBS consistently scores slightly higher than standard beam search. This small improvement in BLEU suggests that MCBS is able to capture better n-gram sequences. The most significant gains are observed in CIDEr, where MCBS steadily improves as the beam size increases, surpassing standard beam search. This suggests that CABS is more effective at generating contextually aligned and relevant captions. Similarly, the METEOR and ROUGE scores for CABS are higher across different beam sizes, indicating that CABS produces captions that are more fluent, semantically rich, and coherent. Overall, these results highlight the strength of MCBS in improving the caption quality compared to the standard approach.

### 5.6. Investigation on Other Datasets

In order to validate our proposed decoding strategy, we first tested its performance on datasets with their own training and testing sets (self-eval), including Flickr8k, Flickr30k [46], and Pascal1K [47]. Then, we performed cross-dataset evaluation on Nocaps [48], IAPR TC-12 (IAPR) [47], and Conceptual Captions (CCs) [49] using models trained on MS COCO (cross-eval). These datasets offer a diverse range of images and captions, ideal for assessing how well the approach handles different visual information and caption complexity. Table 6 shows that our method performs well across these datasets, demonstrating its robustness and accuracy.

The experimental results indicate that MCBS improves the performance over traditional beam search across various datasets. On the Flickr8k and Flickr30k datasets, MCBS showed moderate improvements in BLEU-1 (from 67.5 to 69.4 on Flickr8k and from 64.1 to 65.2 on Flickr30k), while CIDEr scores remained stable or experienced a slight decline (from 62.8 to 63.5 on Flickr8k and from 60.9 to 61.5 on Flickr30k). This suggests that, while MCBS effectively leverages contextual information, further optimization is needed for more complex and diverse contexts. On the smaller Pascal1K dataset, the method demonstrated consistent improvements across metrics, with BLEU-1 rising from 49.4 to 49.8 and CIDEr increasing from 58.4 to 59.7, indicating its capacity to capture contextual information even in limited data scenarios.

Additionally, cross-dataset evaluation was conducted to assess the generalization capability of the model. Models trained on MS COCO were tested on datasets like Nocaps, IAPR TC-12, and Conceptual Captions (CCs). The results of this cross-dataset evaluation demonstrate that, while the model performs well on datasets it has not seen during training, further optimization is needed to handle the differences in visual and linguistic distributions across datasets. For instance, on Nocaps, BLEU-1 improved from 24.5 to 25.2 and CIDEr from 54.7 to 54.9, but the model still faced challenges with unseen object categories. On IAPR TC-12, the model achieved a notable increase, with BLEU-1 rising from 58.7 to 60.2 and CIDEr from 57.3 to 59.2. Similarly, on Conceptual Captions, BLEU-1 improved from 47.2 to 48.3 and CIDEr from 55.3 to 56.8, suggesting that MCBS is more robust to domain shifts compared to the standard beam search method, particularly in handling diverse or noisy data. Overall, the method shows promise in enhancing image captioning by utilizing context, though further refinements may be necessary to maximize its effectiveness in all scenarios.

**Figure 5:**
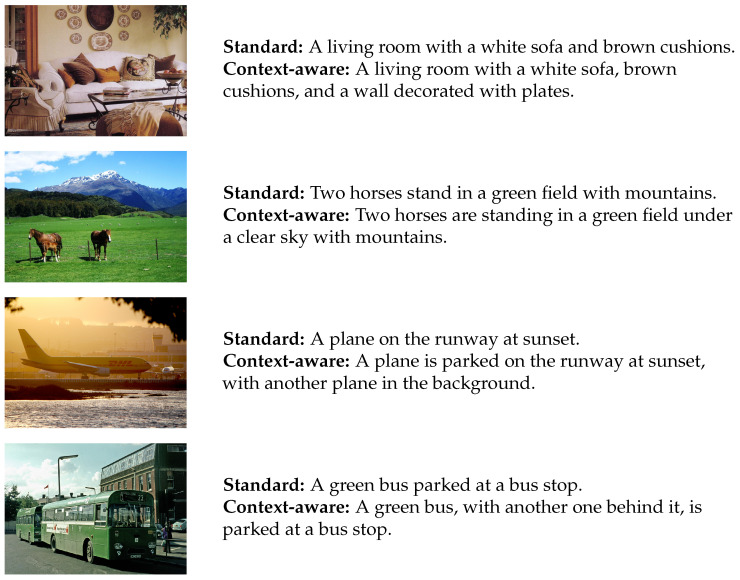
Effectiveness of context-aware beam search on other datasets.

We showcase the text diversity generated by our new decoding strategy across multiple datasets in Figure 5. The examples illustrate how MCBS effectively captures content from different image regions, highlighting CABS’s ability to produce more diverse and nuanced outputs, improving performance in various text generation tasks.

## 6. Conclusions and Future Work

This paper thoroughly reviews caption generation strategies, from greedy search to beam search, and evaluates the performance of image captioning models across different decoding techniques. While numerous beam search variants have been proposed, their application in image captioning remains underexplored. In this study, we not only evaluated these methods but also conducted a detailed comparative analysis. Building upon existing techniques, we introduced the MCBS, specifically designed for image captioning, and demonstrated its effectiveness across multiple datasets and models. Experimental results show that our proposed method significantly improves caption generation quality.

In the future, MCBS holds significant potential in practical applications such as autonomous driving, where precise image descriptions can improve vehicle perception, and medical imaging, where accurate descriptions can assist in diagnosis. Beyond image captioning, MCBS could also be applied to fields like visual question answering and text translation, broadening its impact.

## Figures and Tables

**Table 1:** Comparison of decoding strategies.

Method	Description	Disadvantages
**Greedy** **search**	Selects the word with the highest probability at each step, leading to a single sequence.	Often leads to suboptimal solutions because it does not explore alternative sequences. Results can lack diversity.
**Top-k** **sampling**	Samples the next word from the Top-k most probable words, allowing more diversity.	Although this method increases diversity, it may lead to incoherent sequences, as words might not match well with preceding ones, causing cumulative errors in generation.
**Nucleus** **sampling**	Samples from the smallest set of words whose cumulative probability exceeds p.	This method tends to generate suboptimal results because it does not evaluate multiple candidate sequences in depth, leading to a higher chance of errors compared to beam search.

**Table 2:** Standard beam search vs. meshed context-aware beam search.

Model	Standard						Meshed Context-Aware				
	**B1**	**B4**	**M**	**R**	**C**		**B1**	**B4**	**M**	**R**	**C**
Trans	80.6	38.8	28.6	58.3	126.4		81.9	40.2	29.1	59.1	129.2
$M^{2}$	80.8	39.1	29.1	58.4	128.7		82.0	40.4	29.2	59.2	130.4
AoA	80.2	38.9	29.2	58.8	129.8		82.0	40.5	29.5	59.4	131.4
DLCT	81.4	39.8	29.5	59.1	133.8		82.6	40.6	29.6	59.5	134.7
BPOD	81.5	39.7	30.0	59.5	135.9		82.9	40.5	29.5	59.6	136.4

**Table 3:** Performance of models under different decoding strategies.

Model	Method	Cross Entropy Loss						Reinforcement Learning Loss				
		**B1**	**B4**	**M**	**R**	**C**		**B1**	**B4**	**M**	**R**	**C**
Trans	GS	75.0	33.0	27.1	55.5	110.2		78.4	33.0	27.7	55.9	117.4
	Top-k	40.1	13.3	16.3	28.4	24.1		42.9	13.6	16.8	28.6	31.5
	Top-p	40.3	13.2	16.3	28.8	24.5		43.0	13.4	17.0	28.7	32.0
	SBS	75.0	34.7	27.3	55.8	111.2		80.6	38.8	28.6	58.3	126.4
$M^{2}$	GS	75.2	34.1	27.4	54.9	113.		79.5	34.5	27.7	55.2	120.6
	Top-k	41.5	13.4	16.4	28.9	25.7		42.9	14.2	17.5	29.0	33.6
	Top-p	41.0	13.5	16.9	29.0	25.2		43.3	14.0	17.4	29.7	33.5
	SBS	75.8	35.3	27.6	55.1	113.5		80.8	39.1	29.1	58.4	128.7
AoA	GS	75.4	34.2	27.3	54.6	114.1		79.8	35.0	27.6	56.2	122.3
	Top-k	42.0	13.9	17.0	28.9	27.7		46.0	14.9	17.2	29.6	36.1
	Top-p	42.4	13.8	16.9	28.2	28.2		45.7	14.5	17.6	29.7	37.2
	SBS	76.4	35.5	28.1	57.3	114.6		80.2	38.9	29.2	58.8	129.8
DLCT	GS	76.4	34.9	27.8	55.4	117.1		80.3	34.8	27.8	56.0	125.3
	Top-k	42.5	14.0	17.1	29.2	28.6		45.5	14.6	17.8	30.7	38.5
	Top-p	42.3	13.9	17.3	28.9	28.1		46.2	14.8	17.9	30.7	39.9
	SBS	76.9	36.9	28.3	56.6	117.8		81.4	39.8	29.5	59.1	133.8
BPOD	GS	77.0	34.8	28.0	55.6	117.2		80.3	34.8	27.8	56.0	125.3
	Top-k	43.5	14.1	17.3	29.9	29.6		47.0	15.6	18.7	32.7	40.2
	Top-p	43.2	14.5	16.9	30.2	30.8		47.1	15.4	18.9	32.9	41.3
	SBS	76.9	36.7	28.4	56.6	117.9		81.5	39.7	30.0	59.5	135.9

GS (greedy search), Top-p (nucleus sampling), SBS (standard beam search).

**Table 4:** Performance of models under beam search variation.

Model	Method	Cross-Entropy Loss						Reinforcement Learning Loss				
		**B1**	**B4**	**M**	**R**	**C**		**B1**	**B4**	**M**	**R**	**C**
Trans	SBS	75.0	34.7	27.3	55.8	111.2		80.6	38.8	28.6	58.3	126.4
	DBS	75.7	34.8	27.2	55.7	110.6		80.3	38.4	28.7	58.3	125.7
	LPBS	74.9	34.8	27.3	55.8	111.0		80.7	38.6	28.7	58.4	126.4
	SAPBS	72.6	32.5	26.4	53.4	106.7		78.6	37.3	27.9	57.4	121.4
	LBS	74.9	34.7	27.4	55.7	111.9		80.8	38.9	28.9	58.5	126.6
	MCBS	75.9	34.9	27.5	55.9	112.0		81.9	40.2	29.1	59.1	129.2
$M^{2}$	SBS	75.8	35.3	27.6	55.1	113.5		80.8	39.1	29.1	58.4	128.7
	DBS	76.0	35.2	27.8	55.1	112.8		81.0	39.1	29.1	58.6	128.4
	LPBS	75.9	35.3	27.8	55.0	113.6		80.9	39.4	28.8	58.9	128.9
	SAPBS	73.1	33.5	26.1	53.1	108.5		78.3	38.3	27.4	57.3	123.6
	LBS	76.0	35.4	27.5	55.3	113.8		81.3	39.4	29.0	58.5	129.2
	MCBS	76.2	35.6	27.8	55.2	113.9		82.0	40.4	29.2	59.2	130.4
AoA	SBS	76.4	35.5	28.1	57.3	114.6		80.2	38.9	29.2	58.8	129.8
	DBS	76.5	35.4	28.3	57.0	113.9		81.3	39.0	29.3	58.9	129.5
	LPBS	76.4	35.5	28.5	57.4	114.7		80.1	38.8	29.0	58.8	130.2
	SAPBS	74.7	34.3	27.4	56.4	110.3		78.7	37.5	28.7	57.1	125.3
	LBS	76.5	35.4	28.7	57.5	114.9		80.5	39.2	29.4	59.2	130.4
	MCBS	77.1	35.5	28.8	57.4	115.0		82.0	40.5	29.5	59.4	131.4
DLCT	SBS	76.9	36.9	28.3	56.6	117.8		81.4	39.8	29.5	59.1	133.8
	DBS	76.8	36.4	28.1	56.6	117.4		81.0	39.4	29.4	58.9	133.0
	LPBS	77.0	36.9	28.4	56.8	117.9		81.5	40.0	29.5	59.4	133.7
	SAPBS	73.7	35.1	27.2	54.3	112.5		79.2	38.9	28.9	57.6	129.3
	LBS	76.8	36.9	28.4	56.5	117.9		82.8	40.1	29.8	59.3	130.8
	MCBS	77.2	37.0	28.5	56.7	118.2		82.6	40.6	29.6	59.5	134.7
BPOD	SBS	76.9	36.7	28.4	56.6	117.9		81.5	39.7	30.0	59.5	135.9
	DBS	76.7	36.5	28.4	56.3	117.2		81.5	39.6	29.7	59.3	134.8
	LPBS	77.0	36.5	28.1	56.8	117.8		82.0	39.7	30.5	59.7	135.8
	SAPBS	74.9	35.7	27.6	55.2	113.1		80.3	38.8	29.1	59.2	131.3
	LBS	77.4	37.1	28.1	56.8	118.3		81.7	40.0	30.2	59.4	136.2
	MCBS	77.2	37.1	28.2	56.8	118.6		82.9	40.5	29.5	59.6	136.4

SBS (standard beam search), DBS (diverse beam search), LPBS (length penalty beam search), SAPBS (soft alignment penalty beam search), LBS (lookahead beam search), MCBS (meshed context-aware beam search).

**Table 5:** Model performance using different components.

Trans	Context	Penalty	B1	B4	METEOR	ROUGE	CIDEr
✔			80.6	38.8	28.6	58.3	126.4
✔	✔		81.2	39.9	28.9	58.7	128.7
✔		✔	80.7	38.9	28.5	58.4	126.9
✔	✔	✔	81.9	40.2	29.1	59.1	129.2

**Table 6:** Performance on different datasets.

Eval	Datasets	Standard						Meshed Context-Aware				
		**B1**	**B4**	**M**	**R**	**C**		**B1**	**B4**	**M**	**R**	**C**
Self	Flickr8k	67.5	20.7	22.1	48.2	62.8		69.4	21.2	22.0	48.5	63.5
	Flickr30k	64.1	18.9	21.5	46.3	60.9		65.2	18.9	21.7	46.7	61.5
	Pascal1K	49.4	14.2	18.4	40.5	58.4		49.8	14.5	19.2	41.3	59.7
Cross	Nocaps	24.5	8.1	12.4	24.8	54.7		25.2	8.4	12.6	24.6	54.9
	IAPR	58.7	17.2	20.1	45.7	57.3		60.2	17.8	20.4	46.1	59.2
	CC	47.2	15.9	18.6	40.1	55.3		48.3	16.2	18.9	40.6	56.8

## Data Availability

The MSCOCO dataset is publicly available and can be downloaded from the https://cocodataset.org, accessed on 1 April 2015. The data generated in this experiment can be obtained by contacting the first author or corresponding author.
